# Supplementary material for: What affects power to estimate speciation rate shifts?
Source: PeerJ. 2018 Aug 21;6:e5495. doi: 10.7717/peerj.5495 (PMC6108317; doi:10.7717/peerj.5495)
Supplement: Supplemental Information 1 — Part A provides information on pilot runs of Simulation Set 1, Part B on simulation of replicate trees in Simulation Set 1, Part C on pilot runs of Simulation Set 2 and Part D on simulation of replicate trees in Simulation Set 2 [file peerj-06-5495-s001.pdf]

Supplemental Information 1: Additional details of the simulation procedure

Part A provides information on pilot runs of Simulation Set 1, Part B on simulation of replicate trees in Simulation Set 1, Part C on pilot runs of Simulation Set 2 and Part D on simulation of replicate trees in Simulation Set 2.

A) Pilot runs to choose B_{age} for Simulation Set 1

Initial simulations showed that a λ_0 value of 0.2 along with a μ value of 0.05 allowed us to generate trees with the required parameters, and therefore these values were fixed across all simulations for Set 1. Further pilot runs were conducted using a large range of B_{age} values to find out which values could result in a given target size class for a given combination of relative *subtree* age and speciation rate asymmetry (Table 1, Section 1b). A pilot run involved randomly choosing an age (in this case, B_{age}) between 5 and 35 units, and simulating 1000 trees using the function *tess.sim.age* in the R package TESS. The 1000 trees generated in this way typically varied in size. The function *tess.nTaxa.expected* in the same package gives the expected number of tips for a given speciation rate, extinction rate and tree age. We chose one tree out of the 1000 trees generated in a single run, using correlation tests. The correlation tests were done using the function *cor.test* and involved calculating the correlation coefficient of the expected and simulated numbers of tips at each 1 unit time interval. Because simulated tip numbers were compared with the expected tip numbers, the size of a tree with the highest correlation coefficient best matches the expected tree size. Thus, we computed correlation coefficients for all 1000 simulated trees in a run, and the tree with the highest maximum correlation coefficient was chosen as the tree for that parameter combination. This was designated a *basetree*. For this *basetree*, we calculated the S_{age} for the given relative *subtree* age and λ_1 given the speciation rate asymmetry. We conducted a second run with these parameters to generate 1000 potential *subtrees*. The best among these was chosen using correlation tests, as was done for the *basetree* above. The best *subtree* was was grafted onto the *basetree* following pruning of a randomly chosen *basetree* clade with approximately the same age (S_{age} ±2.5%). This generated a composite tree. If the size of this composite tree was within one of target size classes, this B_{age} was used to generate replicate trees for the combination of parameters (see Section B). The procedure was repeated until a suitable B_{age} and 50 replicate trees were available for all combinations of overall tree size, speciation rate asymmetry and relative *subtree* age.

B) Generating replicate trees for a parameter combination in Simulation Set 1

Once a B_{age} was chosen for a combination of tree size class, relative *subtree* age and speciation rate asymmetry (described in Section A), we simulated 50 replicate trees for each parameter combination. Simulation of a replicate tree involved i) simulation of the *basetree* with the associated λ_0, μ_0 and B_{age} using the function *tess.sim.age* ii) simulation of the *subtree* with the associated λ_1, μ_1 and S_{age} iii) grafting of the *subtree* onto the *basetree*. Simulation of the *subtree* and *basetree* both involved runs generating 1000 trees (using the function *tess.sim.age*) and choosing the best among these using correlation tests as described in Section A.

C) Pilot runs to choose $B_{age,}$ relative subtree *age and λ_0 for Simulation Set 2*

Based on initial simulations, we fixed μ at 0.05 and speciation rate asymmetry at 2X across all simulations. We aimed to generate trees of 5 overall size classes, with three tip-ratios in each of these size classes (Table 1, Section 1c). In order to achieve this, we defined target *basetree* and *subtree* lineage sizes for each overall tree size class. Therefore, the targets of the simulation here were combinations of lineage sizes in the composite trees. Pilot runs were conducted using a large range of B_{age}, relative *subtree* age and λ_0 values to find out which values could result in the target *subtree* and *basetree* lineage sizes (that eventually produced a composite tree with the target overall size;Table 1, Section 1c). Therefore, in this Simulation Set, the lineage sizes of the *subtree* and *basetree* were the targets in the simulation, rather than the size of composite tree *per se*. The protocols for the simulating the *subtree* and *basetree* were similar to that used in Simulation Set 1. A run was first conducted using randomly chosen values of $B_{age,}$ and λ_0 to generate 1000 trees. The best among these 1000 was chosen as a potential *basetree* using correlation tests, as in Section A. For the *subtree*, another run generated 1000 trees with a randomly chosen age (i.e. S_{age}) and the λ_1 value corresponding to 2X speciation rate asymmetry (in relation to λ_0 used in the previous step), with the limitation that the chosen S_{age} corresponded to a relative *subtree* age between 30 and 70% (in relation to the B_{age} used in the previous step). The best among the 1000 trees was chosen as a potential *subtree.* Once the *subtree* was grafted onto the *basetree*, the *subtree* and *basetree* lineage sizes in the composite tree were calculated. If these matched the target lineage sizes, the combination of $B_{age,}$ relative *subtree* age and λ_0 were used to generate 100 replicate trees for that pair of lineage sizes. Section D describes how replicates were generated. The procedure was repeated until 100 replicate trees were available for all target lineage size pairs.

D) Generating replicate trees for a parameter combination in Simulation Set 2

Once a combination of $B_{age,}$ relative *subtree* age and λ_0 were chosen (described in Section C), we simulated 100 replicate trees for each parameter combination. Simulation of the replicates was the same as for Simulation Set 1.
