# Supplementary figures and images for: What affects power to estimate speciation rate shifts?
Source: PeerJ. 2018 Aug 21;6:e5495. doi: 10.7717/peerj.5495 (PMC6108317; doi:10.7717/peerj.5495)

Power (proportion of shifts detected)

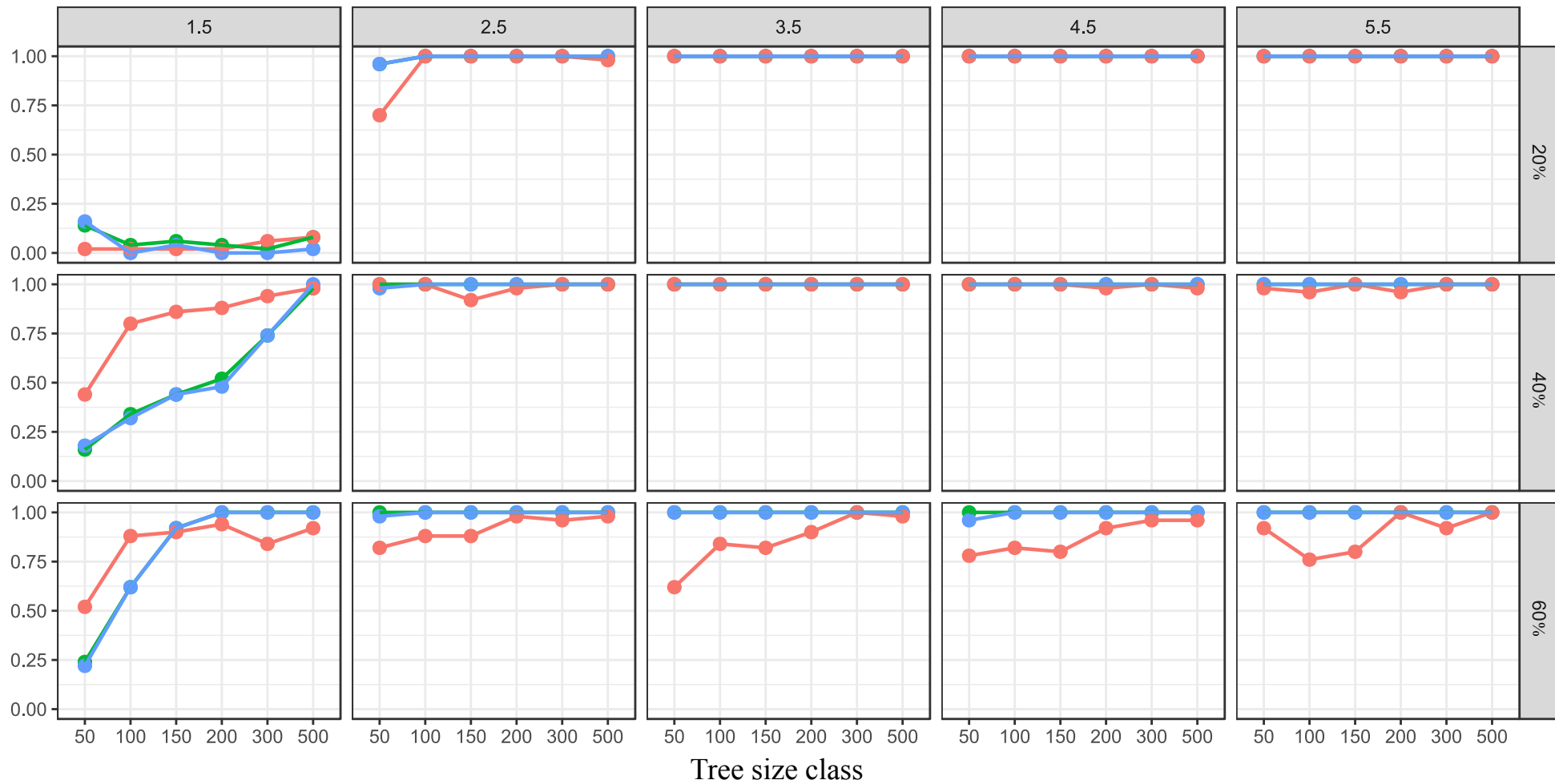

Supplement: Supplemental Information 2 — Variation in ratio of the number of basetree:subtree tips for trees from Simulation Set 1 across the three Sages (A: 20%, B: 40% and C: 60%) and speciation rate asymmetry values (Y axis) and tree size classes (X axis; 50 ± 10, 100 ± 10, 200 ± 10, 400 ± 10 and 800 ± 1). The ratios are colour coded according to the legends [file peerj-06-5495-s002.pdf]

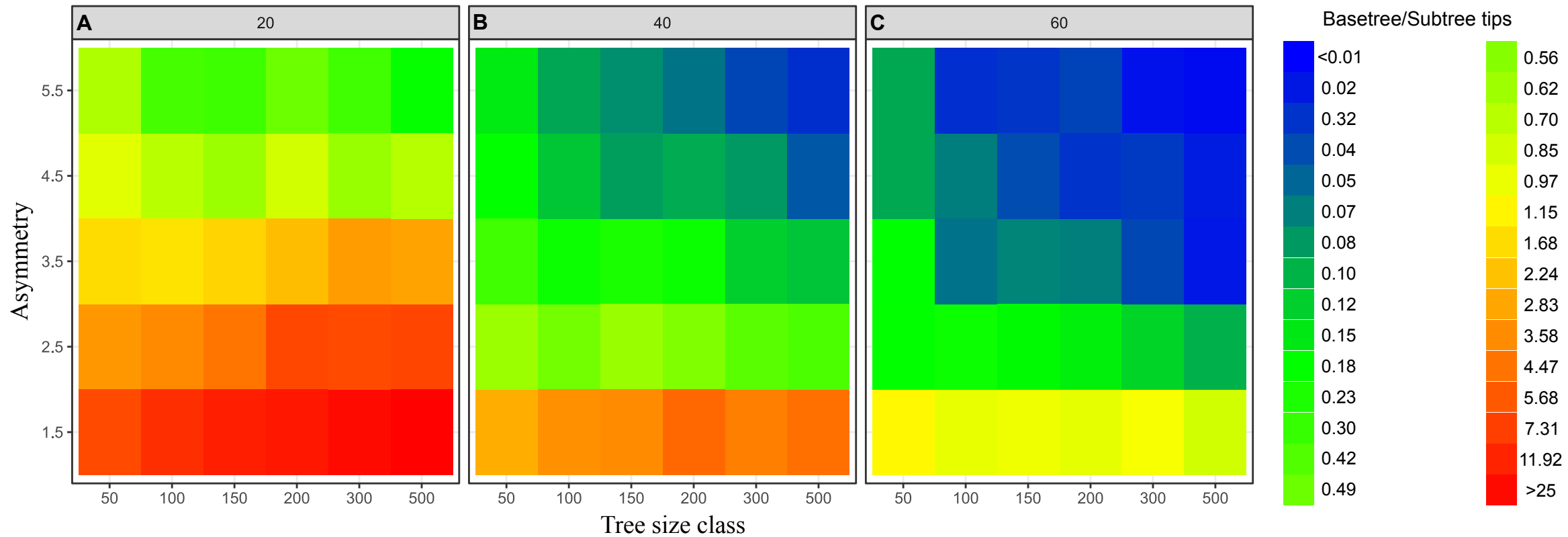

Supplement: Supplemental Information 3 — Comparison of Power of BiSSE, measured as the proportion of shifts detected using the three ‘approaches’ to model selection; red: ‘approach 1’, green: ‘approach 2’ and blue: ‘approach 3’ (see Materials and Methods, Section 2a for details on the differences between the three). [file peerj-06-5495-s003.pdf]
